# Endogenous Two-Photon Excited Fluorescence Provides Label-Free Visualization of the Inflammatory Response in the Rodent Spinal Cord

**DOI:** 10.1155/2015/859084

**Published:** 2015-08-18

**Authors:** Ortrud Uckermann, Roberta Galli, Rudolf Beiermeister, Kerim-Hakan Sitoci-Ficici, Robert Later, Elke Leipnitz, Ales Neuwirth, Triantafyllos Chavakis, Edmund Koch, Gabriele Schackert, Gerald Steiner, Matthias Kirsch

**Affiliations:** ^1^Neurosurgery, Carl Gustav Carus University Hospital, TU Dresden, Fetscherstraße 74, 01307 Dresden, Germany; ^2^Clinical Sensoring and Monitoring, Anesthesiology and Intensive Care Medicine, Carl Gustav Carus University Hospital, TU Dresden, Fetscherstraße 74, 01307 Dresden, Germany; ^3^Centre for Translational Bone, Joint and Soft Tissue Research, Carl Gustav Carus University Hospital, TU Dresden, Fetscherstraße 74, 01307 Dresden, Germany; ^4^Department of Clinical Pathobiochemistry, Carl Gustav Carus University Hospital, TU Dresden, Fetscherstraße 74, 01307 Dresden, Germany; ^5^Center for Regenerative Therapies Dresden (CRTD), TU Dresden, Fetscherstrasse 105, 01307 Dresden, Germany

## Abstract

Activation of CNS resident microglia and invasion of external macrophages plays a central role in spinal cord injuries and diseases. Multiphoton microscopy based on intrinsic tissue properties offers the possibility of label-free imaging and has the potential to be applied in vivo. In this work, we analyzed cellular structures displaying endogenous two-photon excited fluorescence (TPEF) in the pathologic spinal cord. It was compared qualitatively and quantitatively to Iba1 and CD68 immunohistochemical staining in two models: rat spinal cord injury and mouse encephalomyelitis. The extent of tissue damage was retrieved by coherent anti-Stokes Raman scattering (CARS) and second harmonic generation imaging. The pattern of CD68-positive cells representing postinjury activated microglia/macrophages was colocalized to the TPEF signal. Iba1-positive microglia were found in areas lacking any TPEF signal. In peripheral areas of inflammation, we found similar numbers of CD68-positive microglia/macrophages and TPEF-positive structures while the number of Iba1-positive cells was significantly higher. Therefore, we conclude that multiphoton imaging of unstained spinal cord tissue enables retrieving the extent of microglia activation by acquisition of endogenous TPEF. Future application of this technique in vivo will enable monitoring inflammatory responses of the nervous system allowing new insights into degenerative and regenerative processes.

## 1. Introduction

Microglia are maintaining normal cellular homeostasis in the intact spinal cord [1]. They possess a receptor profile that allows the detection of pathogen-related substances as well as substances released by injured neurons and other cell types. Upon pathogen- or injury-related signals, an inflammatory response is elicited in the central nervous system: Microglia cells become activated and transform into an activated phenotype that is characterized by profound changes in cell architecture and morphology. Microglia cells change from a ramified shape with many fine processes towards an amoeboid cell shape bearing any or having only few thick, seldom branched processes. Blood derived macrophages may infiltrate the site of injury after disruption of the blood-brain barrier. They cannot readily be distinguished from activated microglia cells as they share a similar morphology and marker profile [2].

Spinal cord injury (SCI) is causing massive neuronal death and reactive astrogliosis and is accompanied by a temporally and spatially orchestrated inflammatory response. Microglia/macrophages achieve their highest number at five to seven days after injury in the rat model of SCI and persist for weeks [3–5]. In response to SCI macrophages become polarized: They transform into different phenotypes, that is, M1 proinflammatory and M2 anti-inflammatory subset [6, 7]. The role of microglia in SCI is controversially discussed with strong support for a dual role and differential activation [1]. Therefore, visualization of the microglial response to SCI is of fundamental interest for the monitoring and understanding of SCI and any therapeutic intervention. In vivo imaging of SCI in transgenic mice with a fluorescence labeled microglia population was performed [8] and differentiated between resident microglia and invading macrophages in SCI [9].

During the past years, multiphoton imaging techniques addressing intrinsic signals and omitting any kind of external markers or labels were successfully applied to study central nervous tissue pathologies [9, 10]. The combination of coherent anti-Stokes Raman scattering (CARS) that mainly visualizes lipid content, of endogenous two-photon excited fluorescence (TPEF), and of second harmonic generation (SHG) in one image allows the simultaneous assessment of multiple relevant tissue components and structures [11–14]. Spinal cord structure can be resolved down to single axons by imaging myelin sheaths [15], while areas of demyelination can be assessed in inflammatory [16, 17] and traumatic [18, 19] spinal cord pathologies.

Addressing the inflammatory response in SCI in a totally label-free manner seems to be feasible because several reports indicate a marked endogenous fluorescence of microglia/macrophages. Autofluorescence in the cytoplasm of macrophages was found in cases of brain injury by investigation of human autopsy tissue samples [20] and cells displaying intrinsic fluorescence were identified as macrophages/activated microglia in animal models of traumatic brain injury [21]. Irregular large lipofuscin granules in microglia were reported in the main olfactory bulb of young mice [22]. After experimental SCI in a mouse model, infiltration of autofluorescent phagocytic cells into the lesion site was observed [23]. In the injured rat spinal cord, we showed exemplarily that the pattern of endogenous TPEF was linked to the pattern of microglia/macrophages 21 days after injury [11].

In this study, we investigated whether the endogenous fluorescence is a specific marker for activated microglia or a general property of the entire spinal cord microglia/macrophages population. Therefore, subpopulations of microglia/macrophages were identified by immunohistochemistry and compared to structures exhibiting endogenous fluorescence signals at a cellular level at different timepoints after SCI. Furthermore, the relationship of endogenous fluorescence and microglia/macrophages was likewise assessed in a mouse model of experimental autoimmune encephalomyelitis (EAE).

## 2. Methods

### 2.1. Animal Experiments

All animal experiments were performed in accordance with the guidelines of the Dresden University of Technology based on national laws that are in full agreement with the European Union directive on animal experimentation. Surgical procedures were performed under ketamine-xylazine anesthesia, and all efforts were made to minimize suffering. They were approved by the animal welfare committee of Saxony, Germany (Regierungspräsidium, Dresden, Germany, AZ: 24D-9168.11-1-2007-8 and 24-9168.11-1/2010-43).

### 2.2. Experimental Spinal Cord Injury

Experimental spinal cord injury was induced in adult Wistar rats (*n* = 8) by T9-T10 hemisection as described earlier [11, 24].

Briefly, the animals were anesthetized and a skin incision of 2.5 cm was made at the spinal cord segments T8–T10. The laminae were exposed and laminectomy of the 9th and 10th thoracic vertebrae was accomplished. The dura was opened and a 4 mm long longitudinal incision along the midline of the spinal cord was performed. Afterwards, lateral cuts rostral and caudal to the midline incision were made and the isolated hemisection of spinal cord tissue was removed. The autochthone back musculature and the thin superficial muscle layer were sutured.

Spinal cords were harvested 7 and 21 days after hemisection. Four animals were used in each experimental group. Animals were perfusion-fixed using 4% paraformaldehyde (PFA) in phosphate buffered saline (PBS). Spinal cords were removed and postfixed in PFA for 24 h at 4°C. Dehydration in rising sucrose concentration (10% for 24 h and 30% for 24 h) was followed by embedding the isolated spinal cords in tissue freezing medium (Leica, Nussloch, Germany). Samples were frozen on dry ice and stored at −80°C. Cryosections of 16 *μ*m thickness were prepared and stored at −20°C until use.

### 2.3. Experimental Autoimmune Encephalomyelitis

EAE was induced in 9- to 12-week-old female C57BL6 mice (*n* = 6), as previously described [25], by immunization with MOG_35–55_ in emulsion with incomplete Freund's adjuvant in 0.5 mg inactivated* M. tuberculosis* accompanied by an intraperitoneal injection of pertussis toxin on day 0; the latter was repeated on day 2. Mice were sacrificed after 17 days; the mice displayed a score of 2 (hind limb weakness) to 3 (incomplete (one-sided) hindlimb paralysis). Untreated mice served as controls (*n* = 3). After euthanasia, spinal cords were removed, fixed in 4% paraformaldehyde in PBS for 24 h, and embedded in tissue freezing medium (Leica). Samples were frozen on dry ice and stored at −80°C. Cryosections of 16 *μ*m thickness were prepared and stored at −20°C until use.

### 2.4. Histology and Immunohistochemistry

Prior to further processing, sections were allowed to thaw completely and fixed in methanol-acetone (1 : 1) for 10 min at −20°C. To analyze damage after SCI, sections were stained with hematoxylin-eosin according to standard protocol.

For immunohistochemistry heat antigen retrieval in citrate buffer was followed by washing the sections in aqua dest. and PBS. Samples were blocked with 0.1% bovine serum albumin and 3% normal serum in 0.3% Triton X for 1 h and probed with rabbit anti-Iba1 (1 : 200, Wako Chemicals GmbH, Neuss, Germany) that was detected with donkey anti-rabbit AlexaFluor594 (Molecular Probes, Life Technologies GmbH, Darmstadt, Germany). For rat tissue mouse anti-CD68 (1 : 200, Millipore, Merck KGaA, Darmstadt, Germany) and for mouse spinal cord rabbit anti-CD68 (1 : 400 Abcam, Cambridge, UK) antibodies were applied followed by detection with donkey anti-mouse AlexaFluor488 or donkey anti-rabbit AlexaFluor488 (1 : 500, Molecular Probes) overnight at 4°C. The specimens were coverslipped using Vectashield (Vector Laboratories, Burlingame, USA) with DAPI to obtain counterstaining of cell nuclei.

Images were acquired using an upright Axio Examiner Z.1 (Carl Zeiss AG, Jena, Germany) equipped with camera AxioCam. Binary images of the fluorescence signals were calculated in Fiji [26] using the function* color threshold* and the* moments* filter.

### 2.5. Multiphoton Imaging

The system used was described previously [27]. It is equipped with two erbium fiber lasers which have a pulse length of around 1 ps. The laser producing the pump beam (Femto Fiber pro NIR, Toptica Photonics AG, Munich, Germany) emits 780 nm and has a power of 100 mW. The Stokes source used to excite the CARS signal (Femto Fiber pro TNIR, Toptica Photonics AG) was set to 1005 nm (emitted power 1.5 mW), in order to resonantly excite the symmetric stretching vibration of methylene groups at 2850 cm^−1^. The multiphoton microscope is an upright Axio Examiner Z.1 coupled to a scanning module LSM 7 (all from Carl Zeiss AG) and equipped with nondescanned detectors. The excitation light was focused with a C-Apochromat 32x/0.85 objective. All nonlinear signals were excited and acquired simultaneously using the proper optical filtering. The CARS signal was collected in forward direction and filtered using a band pass (BP) filter centered on 647 nm with bandwidth of 57 nm. The SHG signal was acquired in transmission with BP filter centered at 390 nm and bandwidth of 18 nm. The fluorescence signal in the spectral range 500–550 nm was acquired in reflection.

An individual field of view (66 × 132 *μ*m, 220 × 440 pixels) was acquired within 410 ms (high resolution images). For low resolution overview images, individual fields of view of the same dimension consisting of 30 × 60 pixels were acquired within 57 ms. An averaging of 2 or 4 was used. The acquisition of large areas was performed with a tiling procedure; z-stacks were acquired in order to compensate for the lack of planarity of samples and followed by maximum intensity projections to obtain the final images. Acquisition times ranged from 5 min to 15 min.

### 2.6. Quantification of Iba1-, CD68-, and TPEF-Positive Structures

Identical regions were analyzed in the multiphoton images and the corresponding immunohistochemical staining that was performed on the identical tissue section. At two positions of the spinal cord samples ((1) within the lesion and (2) in a peripheral area of white matter showing an inflammatory response at 4 mm distance to the lesion center) three regions of interest were defined and the number of Iba1-, CD68-, and TPEF-positive objects was counted manually. Only objects that could be related to positive DAPI staining were included. All data is expressed as mean ± SEM. Statistical analysis (one-way ANOVA followed by Tukey Multiple Comparison test) was performed using GraphPad Prism 6.0 (GraphPad Software Inc., La Jolla, CA, USA).

## 3. Results

### 3.1. Overall Pattern of TPEF and Microglia Activation after 7 d and 21 d SCI

**Figure 1:**
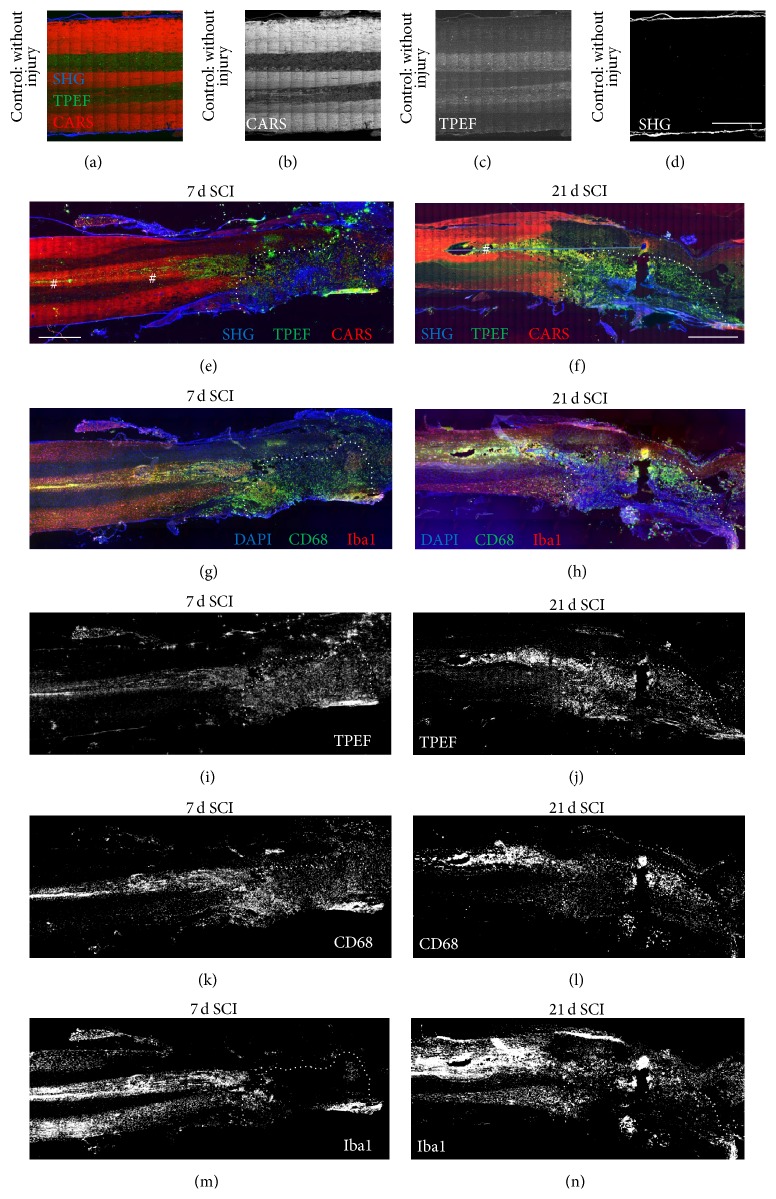
Multiphoton images of spinal cord tissue and immunohistochemistry of microglial markers on the identical section. Control (top) and representative samples 7 d (left) and 21 d (right) after SCI are shown. Images of unlabeled cryosection of spinal cord tissue were obtained by combining second harmonic generation (SHG, blue channel), endogenous two-photon excited fluorescence (TPEF, green channel), and coherent anti-Stokes Raman scattering (CARS, red channel). (a–d) RGB image and single channels of uninjured control spinal cord tissue. (e, f) Multiphoton image of spinal cord 7 d and 21 d after injury. (g, h) Immunohistochemical staining of the same tissue section, overlay of nuclear DAPI staining (blue channel), CD68 (green channel), and Iba1 (red channel). (i–n) Single channel information of endogenous fluorescence and immunohistochemical staining as indicated. The dotted line indicates the region of the lesion. # marks the position of strong endogenous TPEF in peripheral regions. Scale bars: 1 mm.

SHG, TPEF, and CARS were combined in a multimodal image to assess spinal cord morphology. Figures 1(a)–1(d) show the structure of the intact rat spinal cord as reference. Furthermore, multiphoton images of unstained spinal cord tissue sections (*n* = 8) were acquired 7 and 21 days after experimental injury (Figures 1(e) and 1(f)). CARS imaging provides mainly the distribution of lipids within the tissue and visualizes the lipid-rich myelin in high signal intensities. In the multimodal images in Figures 1(a), 1(e), and 1(f), the CARS signal is displayed in red and in Figure 1(b) the single channel information is provided; in the intact spinal cord and distant to the lesion site, alternating white matter tracts (bright CARS signal) and gray matter (low CARS signal) can be recognized in the longitudinal sections. Near and inside the lesion, the intensity of the CARS signal decreases, reflecting a loss of lipids which indicates injury-induced demyelination [11]. In the images in Figures 1(a), 1(e), and 1(f), SHG is displayed in blue; SHG imaging shows the distribution of fibrillar collagen and it provides a visualization of the fibrotic scar in SCI [11]. Intense SHG signal identifies the scar that has developed in the lesion site. In the intact spinal cord, the dura at the external surface of the spinal cords exhibits the most intense SHG signal (Figures 1(a) and 1(d) for single channel). Intense endogenous TPEF (green in Figures 1(a), 1(e), and 1(f)) is observed in the injured spinal cord at the lesion site where also demyelination is detected and extends to peripheral tissue areas in central regions of white matter (as indicated by # in Figures 1(e) and 1(f)).

After multiphoton imaging, immunohistochemistry for microglial markers was performed on the identical tissue section in order to analyze in detail the cellular origin of the endogenous fluorescence (Figures 1(g) and 1(h)). Iba1 (red) and CD68 (green) showed a different expression profile. CD68-positive cells were found in the entire lesion at 7 d after injury and extended in central regions of white matter at both timepoints. Iba1-positive cells were detected in certain parts of the lesion border. Additionally, we observed strong and ubiquitous Iba1 expression in all areas of white matter within the sample.

A thresholding procedure allowed obtaining binary images that clearly illustrate the pattern of the endogenous TPEF signal (Figures 1(i) and 1(j)), of the CD68 (Figures 1(k) and 1(l)), and of the Iba1 immunohistochemistry (Figures 1(m) and 1(n)). The overall pattern of CD68 was in congruence with the pattern of endogenous TPEF that was acquired before staining. The pattern of Iba1 staining extends on larger tissue areas compared to both CD68 and TPEF.

### 3.2. Comparison of TPEF with CD68-Positive and Iba1-Positive Microglia/Macrophages

**Figure 2:**
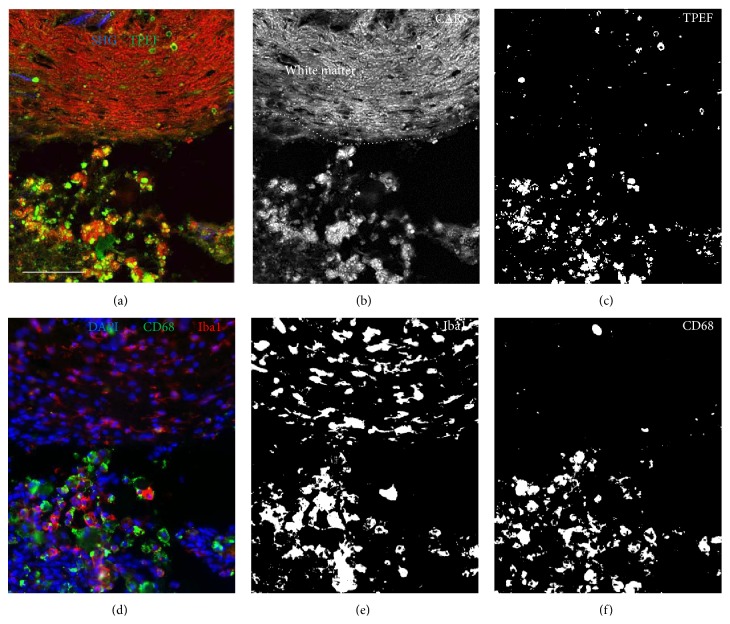
Comparison of endogenous TPEF after SCI and microglial markers at a cellular level. Magnification of the lesion border 21 d after SCI. (a) Multiphoton image of an unlabeled cryosection obtained by combining SHG (blue channel), TPEF (green channel), and CARS (red channel). (b) CARS channel shown in gray scale; the dotted line indicates the border between the strongly damaged tissue area and the more preserved contralateral white matter. (c) The TPEF channel shown after thresholding in black and white. (d) Immunohistochemical staining of the same tissue section as shown in (a–c). Overlay of DAPI staining (blue channel), CD68 (green channel), and Iba1 (red channel). (e) Iba1 immunoreactivity shown in black and white. (f) CD68 immunoreactivity shown in black and white. Scale bar: 100 *μ*m.

Figure 2 shows the lesion border 21 d after SCI to compare label-free multiphoton imaging (Figures 2(a)–2(c)) and the expression of microglial markers (Figures 2(d)–2(f)) on a cellular scale. More preserved tissue areas of white matter were identified by high CARS signal intensity that also enables assessing the disturbed axonal alignment (Figure 2(b), upper part). In these regions, only weak endogenous TPEF was found (Figure 2(c)) and immunohistochemistry revealed Iba1-positive cells with elongated cell bodies (Figure 2(e)) but no CD68-positive cells (Figure 2(f)). In strongly damaged tissue regions (Figure 2(b), lower part), Iba1-positive cells were detected which displayed enlarged cell bodies and a round morphology without processes (Figure 2(e)). Additionally, strong CD68 immunoreactivity and round structures exhibiting intense endogenous TPEF were observed (compare Figures 2(c) and 2(f)). Structures displaying endogenous fluorescence were assigned to originate from CD68-positive microglia/macrophages.

### 3.3. Quantitative Comparison of Endogenous TPEF and Microglial Markers

**Figure 3:**
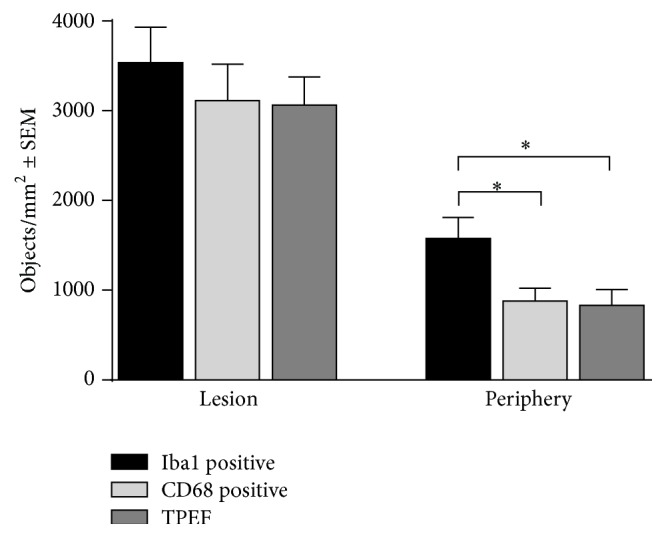
Quantitative analysis of TPEF-positive structures and the expression of microglial markers. The number of Iba1-, CD68-, and TPEF-positive structures was determined within the lesion and in peripheral tissue regions that displayed an inflammatory response. The bars represent mean ± SEM. *∗* indicates *P* < 0.05, one-way ANOVA followed by Tukey Multiple Comparison test.

The number of TPEF-, Iba1-, and CD68-positive structures was determined in different parts of all samples investigated. As the observed patterns were found to be comparable at both timepoints investigated, data of all samples was merged. The results are resumed in Figure 3. We found 3534 ± 397/mm^−2^ Iba1-positive cells at the lesion border. The number of CD68-positive cells and structures exhibiting endogenous TPEF was slightly lower in the same area, being 3111 ± 407/mm^−2^ and 3064 ± 310/mm^−2^, respectively.

The number of microglia/macrophages and objects displaying TPEF was markedly lower in peripheral tissue that displayed an inflammatory response (# in Figures 1(a) and 1(b)). A comparable number of objects displayed TPEF and CD68 immunoreactivity, while significantly more cells were Iba1-positive (CD68: 883 ± 141; TPEF: 833 ± 176; Iba1: 1580 ± 233; *P* < 0.05).

### 3.4. TPEF in Mouse Model of EAE

**Figure 4:**
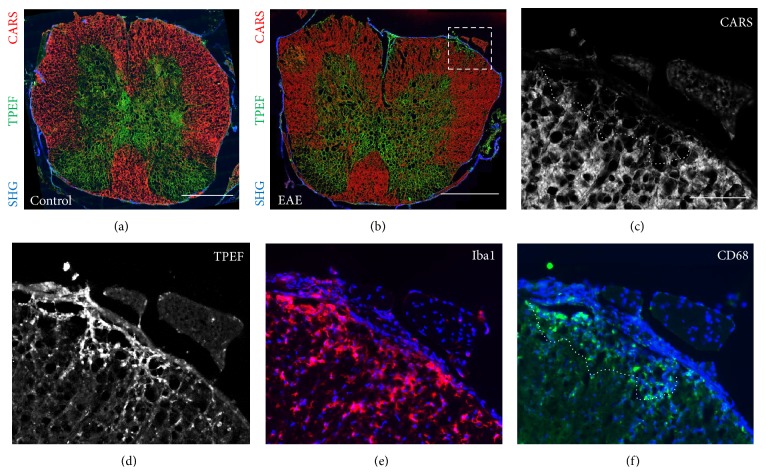
Comparison of endogenous TPEF and microglial markers in a mouse model of experimental autoimmune encephalomyelitis. (a, b) Multiphoton image of an unlabeled cryosection of a control (a) and 17 d EAE (b) mouse spinal cord obtained by combining SHG (blue channel), TPEF (green channel), and CARS (red channel). (c, d) Magnification of the area indicated by the dashed box in (b). (c) CARS channel displayed in gray scale, showing the demyelination of the tissue inside the lesion. (d) TPEF channel displayed in gray scale. (e, f) Immunohistochemistry was performed on consecutive sections. (e) Overlay of DAPI staining (blue channel) and Iba1 (red channel). (f) Overlay of DAPI staining (blue channel) and CD68 (green channel). The dotted line indicates the border of an EAE lesion. Scale bar in (a, b): 500 *μ*m; scale bar in (c–f): 100 *μ*m.

Experimental autoimmune encephalomyelitis (EAE) is a standard mouse model for human multiple sclerosis, which is an inflammatory demyelinating disease of the central nervous system [28]. Therefore, we investigated endogenous TPEF in spinal cords of EAE mice. Figures 4(a) (control) and 4(b) (EAE) show multimodal multiphoton images that were acquired on unstained cross sections. Peripheral areas of white matter tracts display an intense CARS signal and can be clearly discerned from the butterfly-shaped central gray matter. SHG indicates the dura on the spinal cord surface. Figures 4(c) and 4(d) show a zoom-in of the area indicated in Figure 4(b): The typical EAE-induced demyelination can be recognized by diminished CARS signal intensity (Figure 4(c)). Additionally, the EAE lesion (dashed line in Figure 4(b)) is characterized by intense endogenous fluorescence (Figure 4(d)). When comparing the TPEF pattern with CD68 staining (Figure 4(f)), it is possible to observe substantial matching of cellular structures: CD68-positive cells are present in the lesion area only. Iba1-positive cells are more numerous and are present also outside the lesion area (Figure 4(e)).

## 4. Discussion

In accordance with previous studies, label-free multiphoton imaging guaranteed the localization of spinal cord lesions in both models, SCI [11, 18] and EAE [16, 17], respectively. Regular spinal cord structure was disrupted; a decrease in CARS signal intensity clearly indicated demyelination while SHG enabled locating fibrotic scarring. Furthermore, intense endogenous fluorescence related to cellular structures was observed in both models.

The majority of cell autofluorescence in biological tissue originates from mitochondria and lysosomes. The most important endogenous fluorophores are pyridinic (NAD(P)H) and flavins coenzymes, lipofuscins, advanced glycation end products (AGEs), collagen, and elastin (of the extracellular matrix) [29, 30]. The autofluorescence of microglia/macrophage was identified to originate from the redox cofactor, flavin adenine dinucleotide, on the basis of spectral properties [31]. Other authors assigned the endogenous fluorescence to lipofuscin granules by ultrastructural analysis [22].

We already observed in rat SCI a substantial agreement between the punctuated pattern of TPEF and Iba1-positive cells displaying amoeboid round shape that could be interpreted as activated microglia/macrophages [11]. This suggested that the pattern of TPEF provides information about inflammation in SCI models. Here, we refined our research, showing that the pattern of CD68-positive microglia/macrophages resembles the pattern of endogenous TPEF after experimental SCI in the rat. We confirmed these findings down to a cellular level and verified that numbers of CD68-positive cells and TPEF-positive objects are quantitatively comparable. Furthermore, CD68 immunoreactivity and TPEF were confined to certain tissue parts and clearly related to SCI while Iba1-positive cells were dispersed along the entire axis of spinal cord. The number of Iba1-positive cells per unit area was always found to be larger, inside the lesion and especially at the periphery.

The two microglia/macrophages markers used in this study enable visualizing different populations. While Iba1 is a general marker that labels the entire microglia/macrophages population, that is, activated and resting, CD68 is a microglia activation marker [2, 32, 33]. Activated microglia are proliferating nonphagocytic cells that display changes in their immunophenotype and morphology but have not yet transformed into brain phagocytic macrophages [4, 34]. At seven and 70 d after SCI, ~50% of the CD68-positive cells in the lesion were identified as CD8-positive macrophages [5]. They exhibit the classical macrophage morphology of densely packed foamy cells with large lysosomal inclusions and eccentric nuclei [35]. In pathological brain CD68 was shown to label neutrophils as well [36]. The peak of neutrophils after SCI is at postinjury day 3 and the clearance is fast [5]. Therefore, in our experimental setup the cells that were CD68-positive and Iba1-negative in some parts of the lesion 7 d after SCI might represent neutrophils, while 21 d after injury no neutrophils are expected. The maximum number of CD68-positive microglia/macrophages was found 7 d after SCI [4, 5]. A slow clearance of immune cells was described and microglia/macrophages represent the cellular component of sustained inflammation and persist for weeks after injury [2, 5]. This is consistent with the pattern of TPEF we found in this study at 7 d and 21 d after SCI.

TPEF was found in all areas that displayed immunoreactivity for CD68, both in rat SCI and mouse EAE. In SCI, the numbers of TPEF and CD68-positive cells agree very well. The matching at cellular level is also in agreement, considering the inherent difference of the imaging techniques: TPEF is confocal, while fluorescence of immunohistochemical staining was acquired from the whole section thickness. Therefore, the results strongly suggest that the endogenous fluorescence enables identifying activated microglia/macrophages as well as invading neutrophils. The shape and size of the single TPEF-positive object are consistent with the amoeboid morphology of activated microglia/macrophages. We conclude that this is not an artefact resulting from formalin-based fixation as endogenous fluorescence of immune cells/macrophages was already observed on nonfixed tissue and in vivo by other researchers. A similar pattern of endogenous fluorescence has been observed in the lesion after SCI in the mouse on nonfixed tissue sections and was assigned to phagocytes without further immunohistochemical analysis [23]. Furthermore, endogenous TPEF was used to follow immune cell dynamics in the rat cornea in vivo [37] and autofluorescence of invading, likely inflammatory cells, was observed in mouse spinal cord during chronic in vivo imaging using an implanted chamber [38]. The knowledge of the subtype(s) of microglia displaying this endogenous fluorescence would open the possibility for label-free temporal and spatial analyses of the inflammatory response after SCI and in other nervous tissue pathologies in vivo.

## 5. Conclusions

The endogenous fluorescence of cells and tissues is often considered rather as disturbing background or pitfall when analyzing fluorescent staining or expression of fluorescent proteins [21, 31]. In contrast, our data supports the possibility to exploit this endogenous information to retrieve a useful pattern of the inflammation. The pattern of CD68-positive cells representing activated microglia/macrophages was consistent with the pattern of TPEF in rat SCI and mouse EAE. In rat SCI overlay at single cell level was obtained. We envision the application of this approach as a required monitoring tool, possibly usable in vivo, to identify activated microglia and temporal changes of their activity without the need for labels or tissue processing.
